# Mental Nurses in Military Hospitals

**Published:** 1916-09-16

**Authors:** 


### MENTAL NURSES IN MILITARY HOSPITALS.

### A REPLY FROM A CORRESPONDENT.

The letter of "Justice" in The Hospital of August 26, p. 497, raises some interesting points which, in view of editorial comment, become somewhat controversial. As far as one can see, "Justice" merely complains that, in the present dearth of surgical nurses, no consideration is given to the qualifications, such as they are, of a person holding the certificate of the Medico-Psychological Association. She states certain facts which, in her opinion, go to show that the refusal to engage such persons leads to a considerable waste of nursing power. One cannot unreservedly read her plaint as making a "claim of the asylum nurse to be admitted into military hospitals on the same footing as trained general nurses." Such a claim, indeed, is not possible. The Association gives its certificate for proficiency in nursing and attending on insane persons. It is certain that its Council would resent the certificate being used as implying proficiency in any form of nursing other than that which it supervises. At the same time some asylum nurses have the advantage of nursing important surgical cases where, for instance, the asylum staff has surgical proclivities and undertakes necessary operations in the asylum wards. Further, the Association is broad-minded enough to make concessions in examination and in period of training to those candidates who hold a recognised hospital certificate. It might, therefore, be worth the while of the military authorities to take the hint of "Justice," and, going beyond the mere certificate, to make inquiries into the special qualifications of individual applicants.

It is quite true that cut-throats do not abound in asylums; it is equally true that hospital practice is not all splenectomy. In both institutions there is a heavy deadweight of ordinary nursing, partly medical and partly surgical, from which the essentials of sick nursing can be efficiently learned. In all modern asylums at least a fourth part of the space is devoted to the sick and infirm. In addition, the wards for acute cases and for epileptics afford a quota of sick nursing; about one-fourth only is taken up by those chronics who are not likely to call for sick nursing. In addition to this considerable amount of sickness, it must not be forgotten that the mental nurse has an exceptional field for practising preventive nursing. Asthenia, perversion, syphilis, and many other similar agencies afford an immense nidus for the production of disease, which has to be warded off as far as possible by the nurse. The Commissioners not infrequently report that in an asylum containing perhaps two thousand cases no bed-sore exists or has been found *post-mortem*. Many of the patients are general paralytics, or ataxic, or incontinent from senility and so forth. Let us for a moment think of the watching and handling required by each of such patients by night and day, for month after month or year on year; let us also think whether this nursing can be surpassed in its essentials.

But all said and done, each must stick to his own last. It is possible, indeed, that an asylum nurse may be well fitted for general nursing; it is also possible that the general nurse may be fitted for mental nursing. But it would be as hazardous to send a serious surgical case to the seaside with mental nurses as it would be to place an actively suicidal dangerous case in a private house under the care of general nurses.

Is it true, as stated, that the asylum nurse now comes from the servant class or is of an inferior social class in comparison with general nurses? Some women of good position and some women of anything but good position enter either branch of nursing. The question of comparative social position thus becomes one of relative proportion and not one of absolute truth. But let us assume that such an averment is true. Is it one that can be made with advantage to anyone? It may be taken to be one of the chief attributes of a profession that it obliterates all such invidious distinctions by its own influences. How many members of our own or of sister professions, starting from a humble, even a subliminal, point have gone forward, by their profession, to "mingle comfortably" not only with their brethren in highest council, but even with the highest in the land? My own experience of hundreds, I may say thousands, of nurses is that very many indeed of them in time acquire, through their profession, the manners, the habit of thought, the reserve, and the altruism that make the true lady, even though they are debarred from the advantages of birth.

Another question that one can usefully put to oneself is—why does one young woman take to hospital nursing while another seeks the asylum? Is it a matter of pecuniary advantage, or one of liberty and discipline, or one of liking for the particular work, or one of moral and possibly physical courage, or, finally, one of intention to offer the most self-denying service to fellow-creatures? If the latter is the predominant factor, surely the asylum will exercise the greater attraction.

For Matrons' and Sisters' Appointments, see p. 570.
For Complaints of Familiarity, see p. 565.

